# Interaction and potential mechanisms between atorvastatin and voriconazole, agents used to treat dyslipidemia and fungal infections

**DOI:** 10.3389/fphar.2023.1165950

**Published:** 2023-05-11

**Authors:** Tianrong Xun, Yan Rong, Bin Lv, Jinfei Tian, Qing Zhang, Xixiao Yang

**Affiliations:** ^1^ Department of Pharmacy, Shenzhen Hospital, Southern Medical University, Shenzhen, Guangdong, China; ^2^ Department of Pharmacy, Nanfang Hospital, Southern Medical University, Guangzhou, Guangdong, China

**Keywords:** pharmacokinetics, atorvastatin, voriconazole, dyslipidemia, drug–drug interaction

## Abstract

**Purpose:** Voriconazole (VOR) is combined with atorvastatin (ATO) to treat fungal infections in patients with dyslipidemia in clinical practice. However, the pharmacokinetic interactions and potential mechanisms between them are unknown. Therefore, this study aimed to investigate the pharmacokinetic interactions and potential mechanisms between ATO and VOR.

**Patients and methods:** We collected plasma samples from three patients using ATO and VOR. Rats were administered either VOR or normal saline for 6 days, followed by a single dose of 2 mg/kg ATO, and then plasma samples were collected at different time points. The incubation models of human liver microsomes or HepG2 cells were constructed *in vitro*. A high-performance liquid chromatography-tandem mass spectrometry (HPLC-MS/MS) system was developed to determine the concentration of ATO, 2-hydroxy-ATO, 4-hydroxy-ATO, and VOR.

**Results:** In patients, VOR significantly reduced the metabolism of ATO and slowed the formation of 2-hydroxy- and 4-hydroxy-ATO. In rats pretreated with orally administered VOR for 6 days or normal saline given a single dose of 2 mg/kg ATO administered orally on Day 6, the t_1/2_ of ATO was significantly prolonged from 3.61 to 6.43 h, and the area under the concentration-time curve (AUC_0–24h_) values of ATO increased from 53.86 to 176.84 h μg.L^−1^. However, the pharmacokinetic parameters of VOR (20 mg/kg) with or without pretreatment with ATO (2 mg/kg) only slightly changed. *In vitro* studies indicated that VOR inhibited the metabolism of ATO and testosterone, and the IC_50_ values were 45.94 and 49.81 μM. However, no significant change in transporter behaviors of ATO was observed when VOR or transporter inhibitors were co-administered.

**Conclusion:** Our study demonstrated that VOR has significant interactions with ATO, probably due to VOR’s inhibition of the CYP3A4-mediated metabolism of ATO. Based on the clinical cases and potential interactions, the basic data obtained in our study are expected to help adjust the dose of ATO and promote the design of rational dosage regimens for pharmacotherapy for fungal infections in patients with dyslipidemia.

## Introduction

As a competitive inhibitor of 3-hydroxy-3-methylglutaryl coenzyme A (HMG-CoA) reductase, (Lee et al., 2015), atorvastatin (ATO) is a third-generation synthetic statin that has been demonstrated to lead to an effective decrease in serum cholesterol and low-density lipoprotein cholesterol levels (Zhao et al., 2017; Hosseinzadeh et al., 2019). ATO is a safe and well-tolerated lipid-lowering agent compared to other statins and is reported to reduce the morbidity and mortality of cardiovascular disease in patients with coronary heart disease (Indumathi et al., 2017; Grigoropoulou et al., 2019). Currently, ATO is used worldwide in clinical practice for primary and secondary prevention of cardiovascular disease (Satoh et al., 2015; Nenna et al., 2017). As a lipophilic statin, ATO has a poor absolute bioavailability of approximately 14% and a plasma protein-binding ratio of more than 98%. It is often administered as the calcium salt of the active hydroxyl acid form (Borek-Dohalsky et al., 2006). ATO has been reported to be a substrate of small intestine efflux transporters such as P-glycoprotein (P-gp) and is mainly mediated by cytochrome P450 (CYP) 3A in one-phase oxidation biotransformation into two active metabolites: 2-hydroxy-ATO and 4-hydroxy-ATO, which are primarily excreted from bile after treatment (Koytchev et al., 2004; Macwan et al., 2011). Moreover, growing experimental evidence has shown that ATO is an inhibitor of efflux transporters, including P-gp, multi-drug resistance-associated protein 2 (ABCC2), breast cancer resistance protein (ABCG2), and CYP3A (Choi et al., 2008); thus, further research is needed on the pharmacokinetic changes of the substrate drugs when used in combination.

Previous research and observations in our clinical practice have shown that ATO is used in combination with triazole antifungals to treat fungal infections in high-risk patients with dyslipidemia (Shaukat et al., 2003; Hsiao et al., 2016; Ernst et al., 2017), especially in elderly patients. However, an increased risk of rhabdomyolysis has been reported after concomitant use of these agents. For example, studies have indicated that itraconazole significantly increases the area under the concentration-time curve (AUC) and mean peak serum concentration (C_max_) of ATO and its metabolites (Kantola et al., 1998; Mazzu et al., 2000).

Voriconazole (VOR) is a triazole antifungal agent that is widely used in clinical practice and is sometimes co-administered with ATO. Although triazole antifungal agents are poorly metabolized by one-step oxidative biotransformation and are excreted through the kidneys in the urine in the form of prototype drugs (Lass-Florl, 2011), they showed definite inhibitory effects on CYPs and significantly increased systemic exposure to drugs metabolized by CYP3A4, such as fentanyl and diazepam (Saari et al., 2007; Saari et al., 2008). In addition, as a moderate CYP3A inhibitor, fluconazole may lead to rhabdomyolysis in some clinical cases with concomitant use of ATO or simvastatin (Kahri et al., 2005; Franz et al., 2011). VOR is a derivative of fluconazole and has been identified as an inhibitor of CYP3A (Kim et al., 2019), which may be a critical factor in the elevated serum levels of tacrolimus (Imamura et al., 2016). An *in vitro* study showed that VOR is a substrate of CYP3A (Wang et al., 2018); furthermore, Lopez and Tayek (2016) reported a case with symptoms of fatigue, jaundice, and cholestatic hepatitis after a combination of VOR and simvastatin (an inhibitor of CYP3A) and concluded that simvastatin may have decreased the elimination rate of VOR in this patient. These cases strongly indicate that rhabdomyolysis or other adverse drug reactions may occur with concomitant use of VOR and ATO.

However, no study has yet reported the pharmacokinetic interaction between ATO and VOR in either pre-clinical animals or humans. Therefore, this study aimed to investigate the pharmacokinetic interaction between VOR and ATO after oral administration of ATO with triazole antifungal agents. The study was designed to clarify the mechanisms underlying drug–drug interactions between ATO and VOR and obtain the basic data for dose adjustment of ATO that may help link the data of pharmacological analysis with clinical effects, promoting the design of rational dosage regimens and decreasing the incidence of adverse reactions when VOR and ATO are combined.

## Materials and methods

### Chemicals and reagents

ATO calcium standards (purity: 95.3%, No. 100590-201303) and VOR standards (purity: 99.7%, No. 100862-201402) were purchased from the National Institutes for Food and Drug Control (Beijing, China). Lansoprazole (purity ≥98%, No. 103577-45-3) used as an internal standard (IS) was purchased from Shanghai Aladdin Biochemical Technology (Shanghai, China). K_2_HPO_4_, KH_2_PO_4_, and sucrose were obtained from Macklin Biotechnology (Shanghai, China). NADPH solutions (solutions A and B) were purchased from Corning Incorporated (New York, USA). The rat liver microsomes used in the experiment were prepared by a method described in the literature. The acetonitrile and methanol were of high-performance liquid chromatography (HPLC) grade and purchased from Merck (Darmstadt, Germany), and the HPLC-grade formic acid and methyl tert-butylether were purchased from Macklin Biotechnology (Shanghai, China). All other reagents were of analytical grade. The ultrapure water used in the experiments was prepared with a Millipore water purification system. The ATO calcium tablets (20 mg) were purchased from Pfizer Pharmaceutical (New York, USA), and the VOR tablets (50 mg) were supplied by Chengdu Huashen Group (Chengdu, China).

### Patient blood sample collection in clinics

The experimental procedures were approved by the Ethics Committee of Shenzhen Hospital of Southern Medical University, and conducted in accordance with the Declaration of Helsinki (No. NYSZYYEC20180017). Subjects were selected from patients seen in the Department of Critical Care Medicine of Shenzhen Hospital of Southern Medical University, who were diagnosed with fungal infections complicated by cardiovascular and cerebrovascular diseases. Written informed consent was obtained from three patients aged 20–55 years. The collection of human blood samples was divided into two steps: 1) after ATO (20 mg) was used for 3 days (ATO can be taken at 8:00 a.m. and is not affected by meals). The collection time was 0 h, 1 h, 2 h, 12 h, and 24 h after taking ATO. There were five collection time points in total. 2) VOR intravenous schedule: 6 mg/kg, q12 intravenous infusion on day 1; 4 mg/kg, q12 intravenous infusion on days 2–3. ATO was taken at 8:00 a.m. Collection time was 0 h, 1 h, 2 h, 12 h, and 24 h after taking ATO. All blood samples were collected in EDTA anticoagulation tubes, and plasma was prepared by centrifugation at 3000 rpm for 10 min. Blood samples were preserved on ice for further processing; after centrifugation at 4000 rpm for 5 min, plasma samples were collected and stored at −80°C until further processing was performed. The concentrations of ATO or VOR in the plasma samples were determined by liquid chromatography-mass spectrometry (HPLC-MS/MS). All procedures followed the ethical principles of the World Medical Association (WMA) Declaration of Helsinki and were approved by the Medical Ethics Committee of Shenzhen Hospital.

### Animals and drug preparation

Male Sprague–Dawley rats (weighing 210–250 g) used in our experiment were all obtained from the Animal Experiment Center of Southern Hospital of the Southern Medical University (Medical Experimental Animal Number SCXK (yue, 2016-0041). The rats were housed in a clean area; the temperature was controlled at 24°C ± 2°C, and the relative humidity was 55% ± 10%. All the animals were provided with normal feed and drank water freely. The animals were allowed to acclimatize for 2 days prior to the experiments and were fasted overnight for 12 h with free access to water before the experiments. In order to maintain a high degree of consistency with the clinical drugs, commercially available drugs were selected in this study. The ATO and VOR tablets were milled into powder with a mortar, and the corresponding amount was weighed according to the drug content of each medicine. Considering the clinical dosage of each drug and the dose equivalence between rats and human subjects, the corresponding quantity of drug powder was taken and resuspended with normal saline to prepare the drug-containing administration solution before treatment. In our research, the dose of voriconazole (20 mg/kg) and atorvastatin (2 mg/kg) in oral administration in rats was calculated using the formula referred to in previous studies (Nair and Jacob, 2016).

### HPLC-MS/MS analysis of drugs in plasma

Treatment of the blood plasma: 40 µL of blood plasma was mixed with 10 µL of internal standard working solution (500 ng/ml) and acidified with 100 µL CH_3_COONa (0.1 mol/L). After the methyl tert-butyl (1 ml) was added, the mixture was vortexed for 5 min and centrifuged at 3000 r/min for 10 min at 4°C. The supernatant was removed into another 10 ml conical glass tube and dried in a rotary evaporator at 45°C. The residue was redissolved in 100 µL methanol and vortexed for 1 min, followed by HPLC-MS/MS analysis.

A Thermo Scientific™ TSQ Quantiva™ triple-stage quadrupole mass spectrometer (Thermo Fisher Scientific, MA, USA) equipped with a Thermo Scientific™ OptaMax™ NG ion source was used for the experiments. Prelude SPLC™ System samples, pretreatment technology (TurboFlow Eddy current purification), and a Thermo Scientific liquid chromatography system were also connected to the mass spectrometer. The HPLC conditions were as follows: column, Thermo Hypersil Gold C18 column (2.1 × 100 mm, 1.9 μm), column temperature maintained at 40 °C; TurboFlow column, Thermo Cyclone TM-P (0.5 × 50 mm) (ThermoFisher Scientific, MA, USA). The injection volume is 2 μL. A binary gradient mobile phase comprising (A) 0.1% formic acid and (B) acetonitrile was set at 0.4 ml/min, and the gradient elution procedure was as follows: 0–0.43 min, 10% B; 0.43–1.18 min, 10% B→60% B; 1.18–1.68 min, 60% B; 1.68–3.18 min, 60% B→100% B; 3.18–3.85 min, 100% B; 3.85–4.52 min, 100%B→10%B; and 4.52–5.5 min, 10% B.

The analytes were quantified by selective reaction monitoring (SRM) that used heating electrospray ionization (H-ESI) in the positive ion mode. The main working parameters of the mass spectrometry were regulated as follows: ion spray voltage, 3 kV; capillary temperature, 350°C; sheath gas (N_2_) flow, 45 Arb; auxiliary gas (N_2_) flow, 10 Arb; and collision gas (Ar) flow, 2 mTorr. Monitoring results of the precursor ion and product ion were obtained: m/z 559.3→440.2 for ATO, m/z 350.2→281 for VOR, and m/z 370.2→252 for IS. The collision energy of ATO, VOR, and IS was optimized to 21, 16, and 11 eV, respectively. Thermo Scientific™ Pinpoint™ software version 1.3 (ThermoFisher Scientific, MA, USA) was used for data acquisition.

### Pharmacokinetic interaction study in rats

Two separate studies were conducted in this experiment. The Sprague–Dawley rats that participated in Study I were equally randomized and divided into two groups, with six rats in each group. The 6-day oral pretreatment was either 40 mg/kg VOR on Day 1 and 20 mg/kg on days 2–6 or physiological saline (blank group) once daily at 8 a.m. On Day 6, 1 hour (at 9 a.m.) after the last dose of VOR or physiological saline, a single oral dose of ATO (2 mg/kg) was administered orally within 10 min. The rats in each group fasted for at least 1 h before the administration of ATO and were fed a standard meal after 4 h. Next, blood samples (0.2 ml aliquots) were collected from the orbital venous plexus into a heparinized tube before drug administration and 0.17, 0.33, 0.5, 1, 2, 3, 4, 6, 8, 12, and 24 h after oral administration of ATO. There was timely supplementation of physiological saline after the massive blood collection in rats.

The rats in Study II were equally randomized and divided into two groups, with six rats in each group, including physiological saline + VOR (20 mg/kg) and ATO (2 mg/kg) + VOR (20 mg/kg). The blood samples (0.2 ml aliquots) were collected after taking a single dose of the test drug, which was the same as described in Study I. The blood samples were preserved on ice for further processing; after centrifuging at 4000 rpm for 5 min, the plasma samples were obtained and stored at −80°C until further analysis was performed. The concentrations of ATO or VOR in the plasma samples were determined by liquid chromatography-mass spectrometry. Non-compartmental pharmacokinetic analysis of plasma data was conducted using DAS (2.0 version, BioGuider Co., Shanghai, China) to calculate the parameters in the pharmacokinetic study.

### Rat liver microsome isolation

Rat liver microsomes were isolated by standard differential centrifugation procedures according to the method previously described in our study (Wang et al., 2018). Briefly, the pre-chilled phosphate buffer (1.5 mM EDTA, 1 mM DTT, 8 mM KH_2_PO_4_, 0.28 mM PMSF, and 5.6 mM Na_2_HPO_4_) was used to perfuse the liver tissue. Then, the collected tissue was shredded and homogenized in 50 mM phosphate buffer (pH = 7.4, 1 mM EDTA, 0.28 mM PMSF, and 250 mM sucrose). The tissue mixture was centrifuged at 12,000 × g for 15 min at 4°C, and the sediment was discarded. The supernatant was centrifuged at 35,000 × g for 1 h at 4°C. The microsomes were resuspended in 250 mM sucrose. The mixture was immediately stored at −80°C. The concentration of liver microsomes was determined using a BCA protein assay kit (KGSK4051, KeyGEN BioTECH, Jiangsu, China).

### HPLC analysis of 6β-hydroxytestosterone in microsomes

The concentrations of 6β-hydroxytestosterone were determined on a Shimadzu LC-20 AT series HPLC system (Shimadzu Corporation, Kyoto, Japan) by injecting 20 μL aliquots of the processed samples onto an Agilent Eclipse Plus C18 column (4.6 × 250 mm, 5 μm), which was heated in a column oven to 30°C. The isocratic mobile phase consisted of (A) acetonitrile and (B) water (containing 0.1% formic acid) (60:40, v:v) and was delivered at 1 ml/min. The UV detector wavelength was set to 247 nm for ATO and 245 nm for 6β-hydroxytestosterone.

### Effects of VOR on ATO metabolism in microsomes

Rat liver microsomes were prepared to determine the metabolic rate of ATO (Wang et al., 2018). We used testosterone as a probe substrate to evaluate the inhibition of the microsomal CYP3A enzyme activities by determining the formation of 6β-hydroxytestosterone from testosterone and calculating the half-maximal inhibitory concentration (IC_50_) values of VOR. In brief, testosterone was incubated in a reaction system in the presence and absence of relevant concentrations of VOR in rat liver microsomes for a certain time. The concentration of 6β-hydroxytestosterone was measured by an HPLC using the aforementioned method. The effects of VOR on the metabolic rate of ATO were investigated by adding a reaction mixture that comprised 2 μM ATO, 0.4 mg/ml microsomal protein, and 50 mM phosphate reaction buffer (pH 7.4) and pre-incubating in a water bath set at 37°C for 5 min. Next, VOR in the concentration range of 0.39–800 μM was added. Optimization of conditions for ATO incubation was conducted before the experiment began by altering incubation time, microsomal protein levels, and substrate concentrations. The reaction was initiated by the addition of an NADPH-generating system (solution A and B) and incubated at 37°C for 20 min. The incubations were performed in duplicate. The reaction was terminated by placing the tubes on ice and immediately adding 0.2 ml of ice-cold methanol. The resultant clean supernatant (0.2 ml) was collected and analyzed by HPLC for the residual ATO in the reaction system. The percentage metabolism of ATO in the absence and/or presence of VOR was compared, and the difference was taken as an inhibition effect.

### Effects of VOR on ATO efflux transporters in Caco-2 cells

Intracellular accumulation experiment: Cells were inoculated into 24-well plates for 24 h. The cells were exposed to verapamil (50 μM), MK571 (20 μM), Ko143 (10 μM), or vehicle for 24 h, and the culture medium was replaced with fresh medium containing ATO (5 μg/ml) and incubated at 37°C for 90 min. Then, the cells were washed with PBS and lysed with RIPA buffer. The concentration of ATO in the RIPA lysis buffer was determined by HPLC-MS/MS. The concentration of ATO in the experimental group was compared to the control. Monolayer release experiment: HepG2 cells were inoculated at a rate of 4×10^5^ cells/mL into collagen-coated Transwell six-well plates. The culture medium was changed every other day for the first 6 days and then every day for the following 6 days. Hank’s solution (1.5 ml) was added to the polycarbonate chamber side, and 2.5 ml of blank Hank’s solution was added to the upper layer side. The concentration of ATO in the polycarbonate chamber side was determined in the solution.

### Data analysis

The following pharmacokinetic parameters were estimated using DAS (version 2.0, BioGuider Co., Shanghai, China): terminal elimination half-life (t_1/2_); time (T_max_) to C_max_; peak plasma concentration (C_max_); area under the plasma concentration-time curve (AUC) from zero to 24 h (AUC_0–24 h_); AUC from zero to infinity (AUC_0-∞_); total body clearance (CL), and mean residence time (MRT). All experimental data are expressed as mean ± SD. The statistical significance of differences between means in the pharmacokinetic parameters was analyzed using Student’s (two-tailed) *t*-test and, in the case of t_max_, the Wilcoxon signed-rank test. The statistical analysis and the IC_50_ values were evaluated by fitting the data using GraphPad Prism software (version 5.0). A *p*-value of <0.05 was regarded as statistically significant.

## Results

### Method validation for VOR, ATO, and metabolite determination

**FIGURE 1:**
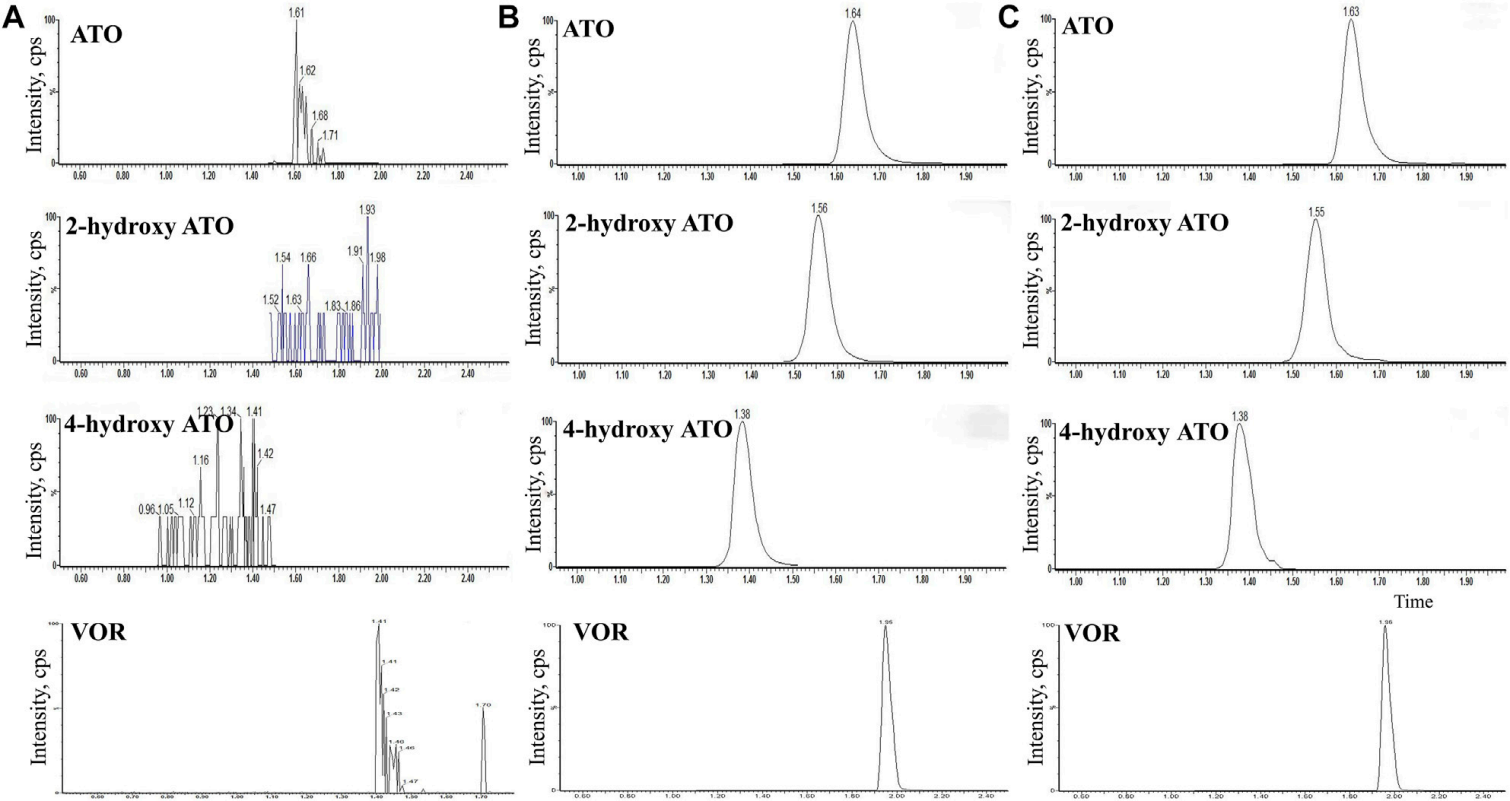
HPLC-MS/MS chromatograms of blank plasma **(A)**, blank plasma spiked with VOR (1000 ng/ml) **(B)**, ATO (200 ng/ml), 2-hydroxy-ATO (200 ng/ml), and 4-hydroxy-ATO (200 ng/ml), **(C)** plasma at 2 h after oral administration of VOR or ATO.

The HPLC-MS/MS method for detecting the concentration of VOR, ATO, and metabolites was validated. Figure 1 depicts representative blank plasma (Figure 1A), blank plasma spiked with VOR (1000 ng/ml), ATO (200 ng/ml), 2-hydroxy-ATO (200 ng/ml), and 4-hydroxy-ATO (200 ng/ml) (Figure 1B), and plasma at 2 h after oral administration of VOR or ATO (Figure 1C). The chromatograms reveal that VOR, ATO, 2-hydroxy-ATO, and 4-hydroxy-ATO were completely separated using HPLC with retention times of 1.95, 1.64, 1.56, and 1.38 min, respectively. The VOR, ATO, 2-hydroxy-ATO, and 4-hydroxy-ATO calibration curves exhibited good linearity and quantitative ranges of 0.01–200 ng/ml. The correlation coefficient was *r* ^2^ > 0.9931. The regression equations were as follows: Y = 0.0092X + 0.4366, Y = 0.0058X + 0.1065, Y = 0.0172X + 0.0020, and Y = 0.0032X + 0.0654, respectively. The intraday precision was less than 9.6%. The interday precision was less than 9.4%. The accuracy was within ±10%. The matrix effects varied from 93.5 ± 5.8% to 101.0 ± 3.0%. The extraction recoveries ranged from 67.9 ± 8.2% to 84.8 ± 4.7%. The long-term stabilities were 2.2%–11.1%, and the three freeze–thaw cycle stabilities were 2.0%–11.5%. These values meet the criteria for the validation of bio-analytical methods.

### Effects of VOR on ATO pharmacokinetics in clinical practice

**FIGURE 2:**
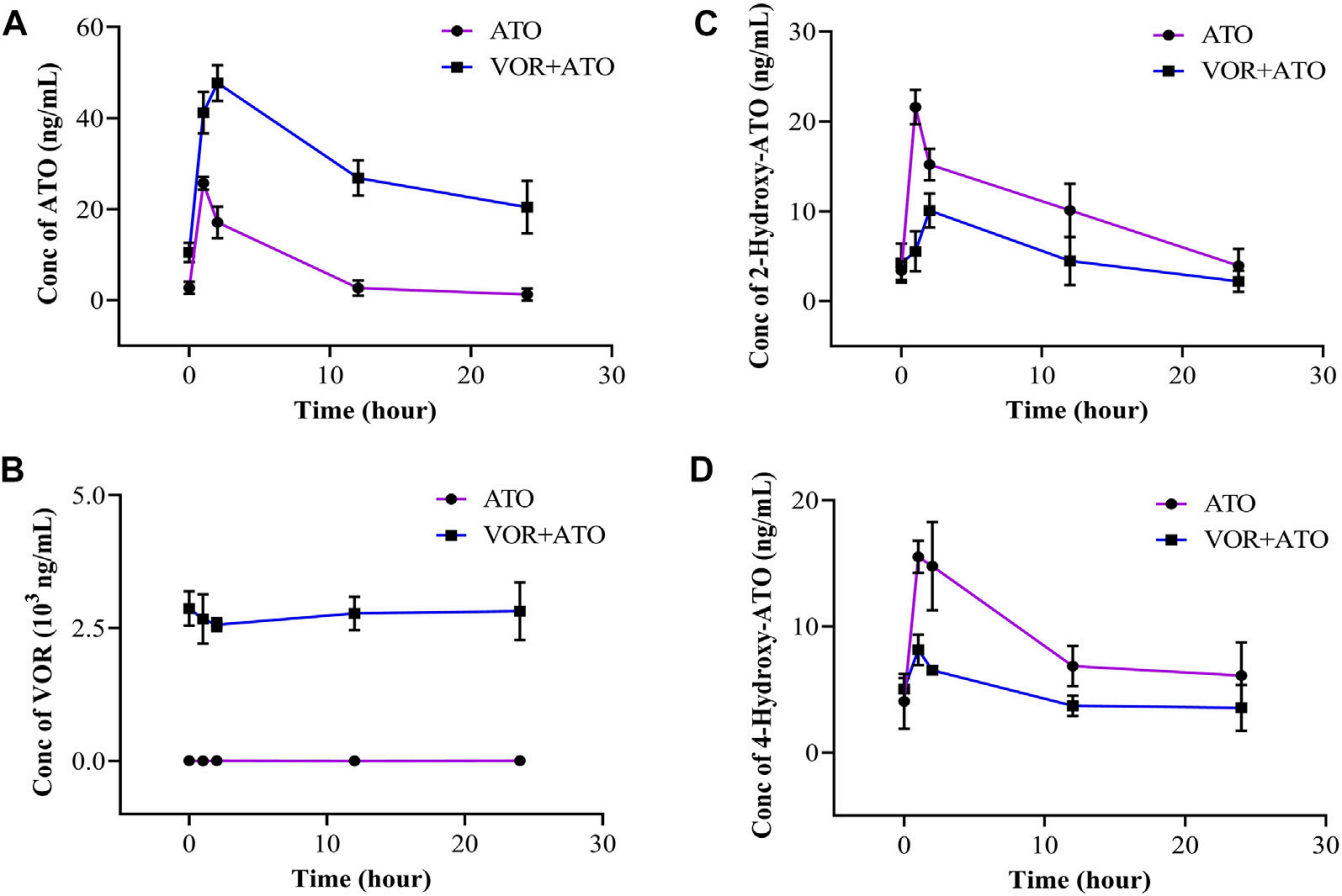
Concentration of ATO **(A)**, VOR **(B)**, 2-hydroxy-ATO **(C)**, and 4-hydroxy-ATO **(D)** in human blood samples following a 3-day pretreatment with voriconazole (6 mg/kg on day 1 and 4 mg/kg on days 2–3) (n = 3).

ATO was used for 3 days, and VOR was administered following the intravenous use program of 6 mg/kg, q12 intravenous infusion on the first day, and 4 mg/kg, q12 intravenous infusion on days 2–3. The plasma concentration versus time profiles of VOR, ATO, 2-hydroxy-ATO, and 4-hydroxy-ATO are shown in Figure 2, and the relevant concentrations are presented in Table 1. These data revealed that the plasma concentrations of ATO, 2-hydroxy-ATO, and 4-hydroxy-ATO were significantly altered in clinical practice after VOR treatment for a few days. The concentrations of ATO in the VOR group were increased by 3.89-, 1.60-, 2.79-, 10.07-, and 16.89-fold after treatment with ATO for 0 h, 1 h, 2 h, 12 h, and 24 h, respectively. However, the metabolites of ATO were significantly reduced. The concentrations of 2-hydroxy-ATO in the VOR group were decreased by 74.34%, 33.53%, 55.72%, and 44.30%, and the concentrations of 4-hydroxy-ATO were decreased by 47.45%, 55.78%, 45.85%, and 41.62% after treatment with ATO for 0 h, 1 h, 2 h, 12 h, and 24 h, respectively. These results revealed that continuous administration of VOR could change the metabolism of ATO in clinical practice.

**TABLE 1:** Concentrations of ATO, 2-hydroxy-ATO, 4-hydroxy-ATO, and VOR in human blood samples following a 6-day pretreatment with VOR (6 mg/kg on day 1 and 4 mg/kg on days 2–3) (n = 3).

Time (h)	ATO		2-Hydroxy-ATO		4-Hydroxy-ATO		VOR	
	ATO	VOR + ATO	ATO	VOR + ATO	ATO	VOR + ATO	ATO	VOR + ATO
**0**	2.697 + 1.342	10.502 + 2.104	3.401 + 1.028	4.239 + 2.160	4.073 + 2.168	5.047 + 0.882	0.007 + 0.012	2.870 + 0.325
**1**	25.710 + 1.426	41.227 + 4.557	21.602 + 1.929	5.543 + 2.235	15.510 + 1.283	8.150 + 1.219	0.000 + 0.000	2.673 + 0.465
**2**	17.093 + 3.491	47.753 + 3.937	15.196 + 1.779	10.100 + 1.896	14.783 + 3.501	6.537 + 0.304	0.007 + 0.012	2.563 + 0.122
**12**	2.663 + 1.678	26.818 + 3.867	10.106 + 2.969	4.475 + 2.687	6.863 + 1.586	3.717 + 0.807	0.000 + 0.000	2.780 + 0.317
**24**	1.210 + 1.326	20.437 + 5.778	3.950 + 1.867	2.200 + 1.184	6.103 + 2.640	3.563 + 1.830	0.007 + 0.012	2.820 + 0.540

### Effects of VOR on ATO pharmacokinetics in rats

**FIGURE 3:**
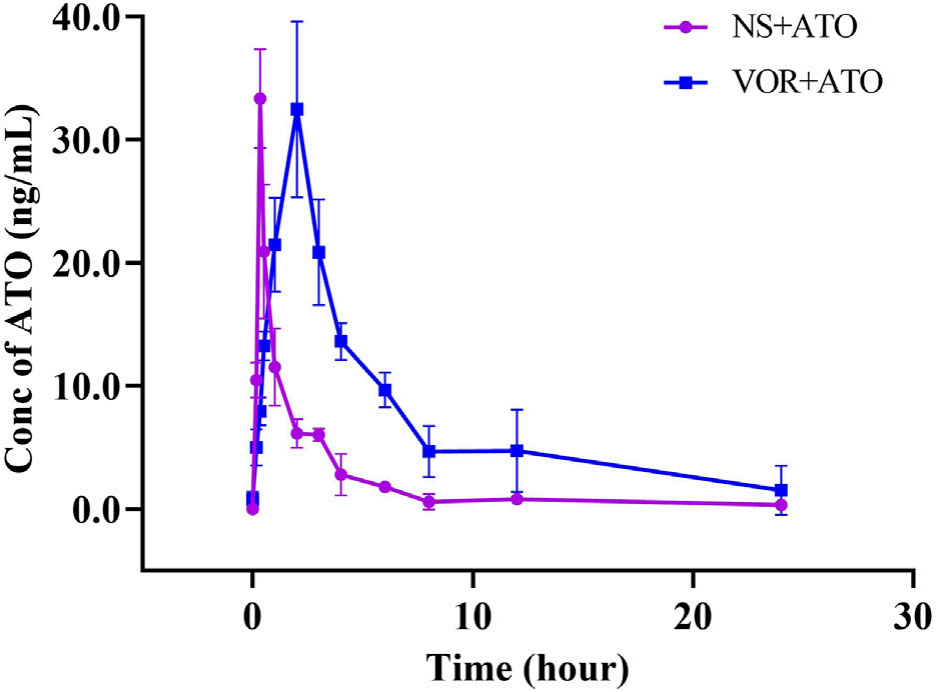
Pharmacokinetic variables of ATO in rats following a 6-day pretreatment with VOR (40 mg/kg on day 1 and 20 mg/kg on days 2–6) or normal saline (NS). The dose of ATO was 2 mg/kg. Data are mean ± SD (n = 6).

The mean plasma concentration-time curves of ATO are presented in Figure 3, and the relevant pharmacokinetic parameters are summarized in Table 2 when ATO (2 mg/kg) was co-administered intragastrically with VOR (40 mg/kg) and blank. The t_1/2_ (6.432 ± 2.094 h), T_max_ (2.000 ± 0.000 h), MRT_0-∞_ (4.172 ± 0.848 h), and mean AUC_0–24h_ (176.848 ± 24.713 h μg.L^−1^) of ATO in the combination of VOR were significantly higher (*p* < 0.05, n = 6) than those of the control rats (3.612 ± 1.151 h, 0.340 ± 0.000 h, 6.730 ± 1.400 h, and 53.863 ± 5.221 h μg.L^−1^); the mean CLz of ATO (0.419 ± 0.955 vs. 4.036 ± 0.003 L h^−1^ kg^−1^) was significantly decreased (*p* < 0.05, n = 6) when ATO and VOR were co-administered. These results demonstrated that the administration of VOR can change the pharmacokinetics of ATO in rats.

**TABLE 2:** Pharmacokinetic variables of ATO in rats following a 6-day pretreatment with VOR (40 mg/kg on day 1 and 20 mg/kg on days 2–6) or normal saline (NS). The dose of ATO was 2 mg/kg. Data are mean ± SD (n = 6), ^*^ *p* < 0.05, compared to the NS group. AUC, area under the concentration–time curve; MRT, mean residence time; CLz/F, total body clearance; t_1/2_, elimination half-life; C_max_, mean peak serum concentration; t_max_, time to reach C_max_.

Parameter	NS + ATO (n = 6)	VOR + ATO (n = 6)
t_1/2_ (h)	3.612 ± 1.151	6.432 ± 2.094*
T_max_ (h)	0.340 ± 0.000	2.000 ± 0.000*
C_max_ (µg.L^−1^)	33.335 ± 4.030	32.493 ± 7.143
AUC_0–24h_ (h.µg.L^−1^)	53.863 ± 5.221	176.848 ± 24.713*
AUC_0-∞_ (h.µg.L^−1^)	55.828 ± 4.537	194.937 ± 47.325*
MRT_0-∞_ (h)	4.172 ± 0.848	6.730 ± 1.400*
Vz/F (L.kg^−1^)	10.189 ± 0.065	2.267 ± 11.024*
CLz/F (L.h^−1^.kg^−1^)	4.036 ± 0.003	0.419 ± 0.955*

### Effects of ATO on VOR pharmacokinetics in rats

**FIGURE 4:**
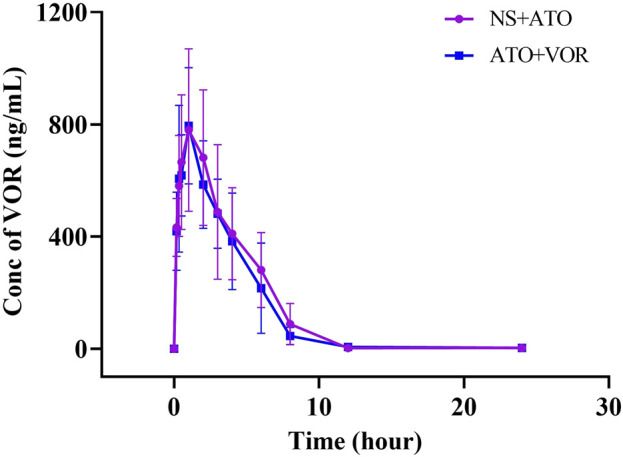
Pharmacokinetic variables of VOR in rats following a 6-day pretreatment with ATO (2 mg/kg) or normal saline (NS). The dose of VOR was 20 mg/kg. Data are mean ± SD (n = 6).

Because the pharmacokinetics of ATO are affected by VOR, the question of whether ATO will affect the pharmacokinetics of VOR arises. The mean plasma concentration–time curves of VOR after oral administration at a dose of 20 mg/kg, with or without ATO (2 mg/kg), are presented in Figure 4, and the relevant pharmacokinetic parameters are summarized in Table 3. The pharmacokinetic parameters of VOR administered with ATO were comparable to those of the control group. Pretreatment with ATO showed no significant difference (*p* < 0.05, n = 6) in the other pharmacokinetic parameters of ATO.

**TABLE 3:** Pharmacokinetic variables of VOR in rats following a 6-day pretreatment with ATO (2 mg/kg) or normal saline (NS). The dose of VOR was 20 mg/kg. Data are mean ± SD (n = 6). AUC, area under the concentration–time curve; MRT, mean residence time; CLz/F, total body clearance; t_1/2_, elimination half-life; C_max_, mean peak serum concentration; t_max_, time to reach C_max_.

Parameter	NS + VOR (n = 6)	ATO + VOR (n = 6)
t_1/2_ (h)	2.582 ± 0.317	2.600 ± 0.419
T_max_ (h)	1.250 ± 0.612	0.917 ± 0.204
C_max_ (µg.L^−1^)	828.525 ± 246.024	826.395 ± 165.775
AUC_0–24h_ (h.µg.L^−1^)	3629.943 ± 802.326	3254.885 ± 861.771
AUC_0-∞_ (h.µg.L^−1^)	3643.294 ± 798.469	3280.268 ± 835.304
MRT_0-∞_ (h)	3.527 ± 0.660	3.547 ± 0.810
Vz/F (L.kg^−1^)	0.022 ± 0.007	0.042 ± 0.041
CLz/F (L.h^−1^.kg^−1^)	0.006 ± 0.001	0.006 ± 0.002

### Inhibitory effects of VOR on the metabolism of ATO in rat liver microsomes and in HepG2 cells

**FIGURE 5:**
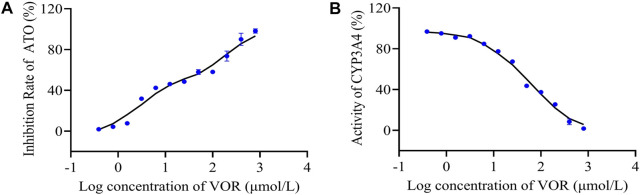
Inhibitory effects of VOR on CYP3A activity in rat liver microsomes. The percentage metabolism of ATO in the absence and/or presence of VOR was compared, and the difference was taken as the inhibitory effect **(A)**. Testosterone was used as a probe substrate of the CYP3A enzyme to assess the inhibitory activities by measuring the formation of 6β-hydroxytestosterone from testosterone **(B)**. Results are expressed as the percent of the normal saline group.

**FIGURE 6:**
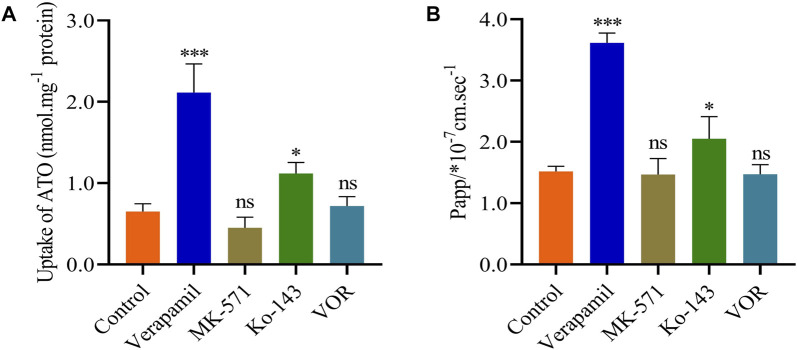
VOR does not affect the pharmacokinetics of ATO through the P-gp, ABCC2, or ABCG2 transporters. Effects of VOR and various inhibitors on the uptake **(A)** and transport **(B)** of ATO. Data are mean ± SD (*n* = 6). ^***^ *p* < 0.001, ^*^ *p* < 0.05, ^ns^ *p* > 0.05 compared to control.

The effects of VOR on ATO metabolism in microsomes *in vitro* are presented in Figure 5A. The inhibitory effects of various concentrations of VOR on the formation of 6β-hydroxytestosterone are presented in Figure 5B. The results obtained from the *in vitro* metabolism study of ATO indicated that VOR could significantly decelerate the metabolism of ATO in rat liver microsomes, and the mean IC_50_ values were 45.94 and 49.81 μM, respectively. However, no significant change in transporter behaviors (Figure 6) of ATO was observed when VOR or transporter inhibitors were co-administered.

## Discussion

With the increasing number of accompanying diseases, prescribing more than one drug for comprehensive therapy is becoming increasingly common in current clinical practice (Lopez and Tayek, 2016). To overcome fungal infections quickly and effectively, drug combinations are important for patients with fungal infections and dyslipidemia. The literature reported that 1.5% of the patients who used statins from October 2001 to September 2002, of which ATO was the most commonly used statin, were also treated with triazole antifungal agents (Liu et al., 2018). However, the combination of drugs may trigger drug–drug interactions and result in adverse drug reactions. The potential for drug interactions may be increased by multi-drug combination therapy mainly because of the presence of similar metabolic pathways or cellular transport pathways between these drugs, which have been demonstrated in pharmacokinetics or pharmacodynamics. Although no more data are available to demonstrate the combined use of triazole antifungal agents and statins, cases of serious adverse reactions caused by combination therapy have been reported. Furthermore, triazole antifungal agents such as VOR and fluconazole may be co-prescribed with ATO in clinical practice. Because VOR tends to require the maintenance of a steady-state concentration and the clinical treatment regimen consists of twice-a-day administration with a doubled first dose, VOR is often pretreated before patients take ATO. Therefore, this study aimed to investigate the pharmacokinetic interaction between VOR and ATO after oral administration of ATO with triazole antifungal agents.

According to the dosing regimens of VOR and ATO and related guidelines, concurrent administration of VOR and ATO requires a special dosing regimen in clinical practice. In our study, VOR was administered for 6 days (40 mg/kg on day 1 and 20 mg/kg on days 2–6), and on day 6, a single dose of 2 mg/kg ATO was administered orally in rats. In patients, VOR significantly reduced the metabolism of ATO and slowed the formation of 2-hydroxy-ATO and 4-hydroxy-ATO. In rats, the t_1/2_ of ATO is significantly prolonged from 3.61 to 6.43 h, and the AUC_0–24h_ values of ATO increased from 53.86 to 176.84 h μg L^−1^. However, the pharmacokinetic parameters of VOR with or without pretreatment with ATO were only slightly altered. The bioavailability of ATO is restricted by a bioavailability barrier composed of intestinal efflux transporters and drug-metabolizing enzymes (Liu et al., 2017), in which CYP3A and P-gp have been recognized as synergistic barriers that affect the bioavailability of ATO. Concerning the dual inhibitor of CYP3A and P-gp, studies have shown that itraconazole has a significant interaction with ATO and that the concomitant use of itraconazole with ATO should be avoided or carefully considered (Stang et al., 2007). However, from the available literature and data, the effects of VOR and fluconazole, which are triazoles, on intestinal efflux transporters are unclear or not obvious (Dong et al., 2008; Guo et al., 2019), and further validation is warranted. Considering that rhabdomyolysis has been reported in current medical practice after the concomitant use of fluconazole and ATO, more attention should be paid to the risk of myopathy during the concurrent administration of VOR with ATO. If results are confirmed in clinical trials, the dose of ATO should be adjusted when ATO is co-administered with VOR.

In contrast, the pharmacokinetics of VOR was not significantly affected by the presence of ATO in our results. Both ATO and VOR were metabolized in the liver and could be competitively inhibited based on hepatic enzyme metabolism (Gupta et al., 2007). However, studies suggested that ATO may be a weak inhibitor of CYP3A with little effect on the pharmacokinetics of drugs metabolized by CYP3A, such as nifedipine (Zhang et al., 2012; Hohmann et al., 2016). Therefore, the pharmacokinetic characteristics of VOR in rats were not significantly altered in combination with ATO in this experiment.

Cytochrome P450 3A4 (CYP3A4) is found in approximately 30% of all hepatic metabolic enzymes in ordinary people, and it is involved in the metabolism of approximately 50% of therapeutic drugs in clinical treatment (Patel et al., 2016). If the activity of CYP3A4 is inhibited, the levels of a concomitantly administered drug in plasma will increase, and the incidence of adverse reactions will increase significantly in clinical settings (Neuvonen, 2010). As we all know, clinical or animal results need to be supported by basic experiments. To further explore whether the effect of VOR on the pharmacokinetics of ATO is related to the inhibition of CYP3A4, we assessed the activity of CYP3A4 *in vitro*. We developed a liver microsomal model to elucidate the interference events. First, we evaluated the metabolism of ATO by CYP3A4 in the liver microsomal model, which showed an inhibitory effect of VOR on the biotransformation of ATO with an IC_50_ value of 45.94 μM. Subsequently, we chose testosterone as the substrate drug of CYP3A4 for further validation. The metabolic enzyme curves were constructed using the inhibition ratio for the VOR-mediated testosterone 6β-hydroxylation activity to clarify the activity of CYP3A4 in an enzyme metabolic system with different VOR concentrations *in vitro*. The results showed that VOR non-competitively inhibited CYP3A4 with an IC_50_ value of 49.81 μM, which is very close to the previous studies. Thus, the mechanism underlying the interaction between VOR and ATO could be because of the inhibition of CYP3A-mediated biotransformation during the first-pass and elimination phases, whereas the inhibitory effect is weaker than itraconazole, consistent with results reported in the literature (Wang et al., 2002). This result further confirmed that continuous administration of VOR can affect the pharmacokinetics of ATO. Furthermore, these results indicate that VOR affects the metabolism of ATO and increases the adverse effects by inhibiting the activity of CYP3A4.

As a bioavailability barrier composed of intestinal efflux, P-gp restricted the elimination of substrate and its metabolites *in vivo* (Liu et al., 2017; Wang et al., 2023). ATO has been reported as a substrate of small intestine P-gp, which is primarily excreted from bile after treatment (Koytchev et al., 2004; Macwan et al., 2011). However, whether VOR affects the pharmacokinetics of ATO via the efflux transporters requires further evidence. Consequently, in the cell transport models, verapamil, MK571, and Ko143, which are transport inhibitors of P-gp, ABCC2, and ABCG2, reversed the efflux of P-gp, ABCC2, and ABCG2, respectively, confirming the contribution of VOR to the efflux of P-gp, ABCC2, and ABCG2. The results showed that VOR had no effect on the activity of P-gp, ABCC2, and ABCG2. Furthermore, these results indicate that the influence of VOR on the pharmacokinetics of ATO may not be achieved by efflux transporters.

The present study has several limitations. First, because most of the patients in the Critical Care Medicine Department receive more drugs than voriconazole and atorvastatin, the interaction between the drugs affected the inclusion of the cases. The sample size for the patient component of our study was three and may not be representative of the entire population. Therefore, the interactions between voriconazole and atorvastatin in clinical practice need further investigation. Second, to the best of our knowledge, this study only analyzed the effects of voriconazole on the activity of several metabolic or transport mediators. Furthermore, this mechanistic study did not analyze the correlation of voriconazole with the presence of metabolic or transport mediators from a molecular biology perspective. We also did not examine the efficacy, adverse effects, or safety caused by the interaction between VOR and ATO. Further studies are needed to determine whether the metabolism of ATO is associated with CYP3A4 or P-gp when patients receive treatment for fungal infections combined with dyslipidemia pharmacotherapy and whether VOR could affect the efficacy and safety of ATO in clinical practice.

The present study demonstrated that VOR has a significant interaction with ATO, probably because of the inhibition of the CYP3A4-mediated metabolism of ATO by VOR. Our findings imply that it is necessary to monitor the concentration of ATO and its metabolites in plasma for patients receiving concurrent voriconazole treatment to reduce the occurrence of adverse reactions to ATO.

## Data Availability

The original contributions presented in the study are included in the article/Supplementary Material; further inquiries can be directed to the corresponding author.
