# Supplementary material for: Interaction and potential mechanisms between atorvastatin and voriconazole, agents used to treat dyslipidemia and fungal infections
Source: Front Pharmacol. 2023 May 11;14:1165950. doi: 10.3389/fphar.2023.1165950 (PMC10213937; doi:10.3389/fphar.2023.1165950)
Supplement: Supplementary file 1 [file Table1.DOCX]

# Supplementary material

**Table 1.** Intra-day and inter-day precision and accuracy of VOR, ATO, 2-hydroxy-ATO,and 4-hydroxy-ATO in rat blood plasma.

| Compound | Nominal concentration  (ng/mL) | Inter-day (n = 5) | | | Intra-day (n = 5) | | |
| --- | --- | --- | --- | --- | --- | --- | --- |
|  |  | Measured  (mean ± SD, ng/mL) | %RE | %RSD | Measured  (mean ± SD, ng/mL) | %RE | %RSD |
| VOR | 0.05 | 0.049±0.002 | -2.47 | 6.89 | 0.047±0.005 | -5.62 | 9.55 |
|  | 5 | 5.103±0.264 | 6.33 | 8.02 | 4.884±0.364 | -3.01 | 5.69 |
|  | 80 | 80.116±4.391 | 8.03 | 4.17 | 81.331±3.654 | 5.23 | 6.72 |
| ATO | 0.05 | 0.046±0.008 | -5.88 | 9.12 | 0.046±0.007 | -9.11 | 3.10 |
|  | 5 | 4.689±0.339 | -5.06 | 9.40 | 4.586±0.662 | -8.03 | 8.36 |
|  | 80 | 78.369±6.223 | -5.17 | 7.05 | 76.131±4.338 | -5.31 | 6.01 |
| 2-hydroxy-ATO | 0.05 | 0.047±0.002 | -3.2 | 5.72 | 0.045±0.006 | -2.92 | 5.12 |
|  | 5 | 4.361±0.487 | -3.87 | 3.02 | 4.755±0.396 | -4.89 | 8.01 |
|  | 80 | 77.589±3.353 | -6.25 | 4.07 | 77.047±5.445 | -9.69 | 6.59 |
| 4-hydroxy-ATO | 0.05 | 0.044±0.006 | -8.9 | 7.12 | 0.047±0.005 | -8.65 | 5.22 |
|  | 5 | 4.551±0.689 | -8.37 | 9.23 | 4.602±0.569 | -8.87 | 8.03 |
|  | 80 | 74.335±7.343 | -9.66 | 8.02 | 74.005±6.584 | -8.56 | 7.55 |

**Table 2.** Matrix effect and extraction recovery of VOR, ATO, 2-hydroxy-ATO,and 4-hydroxy-ATO in rat blood plasma.

| Compound | Nominal concentration  (ng/mL) | Matrix effect (n = 5) | | Extraction recovery (n = 5) | |
| --- | --- | --- | --- | --- | --- |
|  |  | Measured  (mean ± SD, %) | %RSD | Measured  (mean ± SD, %) | %RSD |
| VOR | 0.05 | 96.735±5.337 | 5.25 | 78.032±5.033 | 8.12 |
|  | 5 | 98.446±4.238 | 7.58 | 74.085±5.324 | 5.22 |
|  | 80 | 93.776±9.379 | 3.01 | 84.235±5.002 | 3.78 |
| ATO | 0.05 | 98.117±6.668 | 6.08 | 71.045±7.002 | 8.15 |
|  | 5 | 101.05±3.009 | 3.02 | 73.661±5.671 | 8.03 |
|  | 80 | 93.561±5.884 | 8.01 | 67.881±8.157 | 6.02 |
| 2-hydroxy-ATO | 0.05 | 95.061±4.074 | 8.25 | 74.061±5.069 | 9.58 |
|  | 5 | 94.358±7.480 | 9.23 | 84.775±4.690 | 6.83 |
|  | 80 | 95.451±6.182 | 8.11 | 71.220±5.587 | 6.58 |
| 4-hydroxy-ATO | 0.05 | 98.540±7.001 | 7.45 | 80.112±4.587 | 5.10 |
|  | 5 | 94.556±8.84 | 9.02 | 74.337±9.601 | 8.33 |
|  | 80 | 95.112±6.115 | 8.05 | 70.551±5.545 | 8.52 |

**Table 3.** Stability of VOR, ATO, 2-hydroxy-ATO,and 4-hydroxy-ATO in rat blood plasma.

| Compound | Nominal concentration  (ng/mL) | 24 h at 25℃ (n = 5) | | | Three freeze-thaw cycle (−80℃/25℃) (n = 5) | | |
| --- | --- | --- | --- | --- | --- | --- | --- |
|  |  | Measured  (mean ± SD, ng/mL) | %RE | %RSD | Measured  (mean ± SD, ng/mL) | %RE | %RSD |
| VOR | 0.05 | 0.048±0.004 | -3.55 | 2.21 | 0.045±0.004 | -8.36 | 9.08 |
|  | 5 | 4.962±0.301 | 4.32 | 5.03 | 4.558±0.318 | -7.03 | 5.08 |
|  | 80 | 79.264±5.131 | 5.12 | 3.01 | 75.238±5.338 | 1.58 | 5.17 |
| ATO | 0.05 | 0.047±0.005 | -4.94 | 5.72 | 0.044±0.007 | -9.85 | 10.45 |
|  | 5 | 4.773±0.423 | -4.32 | 3.23 | 4.784±0.357 | -5.14 | 7.46 |
|  | 80 | 77.681±6.004 | -4.25 | 7.55 | 73.885±9.852 | -10.45 | 11.52 |
| 2-hydroxy-ATO | 0.05 | 0.047±0.005 | -0.35 | 11.14 | 0.044±0.004 | -8.35 | 7.56 |
|  | 5 | 4.538±0.424 | -8.25 | 7.25 | 4.554±0.568 | -1.35 | 3.81 |
|  | 80 | 76.114±5.314 | -6.25 | 4.27 | 72.580±4.221 | -10.28 | 9.65 |
| 4-hydroxy-ATO | 0.05 | 0.045±0.007 | -7.78 | 7.57 | 0.044±0.005 | -9.87 | 10.77 |
|  | 5 | 4.625±0.337 | -2.31 | 4.55 | 4.501±0.411 | -1.25 | 2.08 |
|  | 80 | 73.581±6.587 | -9.07 | 8.87 | 72.334±7.338 | -9.69 | 9.88 |
